# Cardiac Differentiation of Pluripotent Stem Cells

**DOI:** 10.4061/2011/383709

**Published:** 2011-04-04

**Authors:** Kristiina Rajala, Mari Pekkanen-Mattila, Katriina Aalto-Setälä

**Affiliations:** ^1^Regea - Institute for Regenerative Medicine, University of Tampere, Tampere University Hospital, 33520 Tampere, Finland; ^2^Heart Center, Tampere University Hospital, 33520 Tampere, Finland

## Abstract

The ability of human pluripotent stem cells to differentiate towards the cardiac lineage has attracted significant interest, initially with a strong focus on regenerative medicine. The ultimate goal to repair the heart by cardiomyocyte replacement has, however, proven challenging. Human cardiac differentiation has been difficult to control, but methods are improving, and the process, to a certain extent, can be manipulated and directed. The stem cell-derived cardiomyocytes described to date exhibit rather immature functional and structural characteristics compared to adult cardiomyocytes. Thus, a future challenge will be to develop strategies to reach a higher degree of cardiomyocyte maturation *in vitro*, to isolate cardiomyocytes from the heterogeneous pool of differentiating cells, as well as to guide the differentiation into the desired subtype, that is, ventricular, atrial, and pacemaker cells. In this paper, we will discuss the strategies for the generation of cardiomyocytes from pluripotent stem cells and their characteristics, as well as highlight some applications for the cells.

## 1. Introduction

Human cardiomyocytes can be isolated from heart biopsies, but the access to human heart tissue is very limited, and the procedure is complicated; it is difficult to obtain viable cell preparations in large quantities, and the cells obtained do not beat spontaneously. Thus, physiologically relevant *in vitro* models for human cardiomyocytes are currently limited. This has led in the creation of alternative models, such as isolation of cardiomyocytes from various newborn animals or production of genetically engineered cell lines overexpressing certain target proteins (e.g., ion channels) [1]. All of these models, however, share significant limitations with respect to their basic physiological differences compared to human cardiomyocytes as well as high costs and ethical questions. A number of different human tissues have been proposed as the source of stem cells able to generate new cardiomyocytes (e.g., fetal cardiomyocytes, adult cardiac progenitor cells, skeletal myoblasts, bone marrow-derived stem cells, adipose-derived stem cells, umbilical cord-derived stem cells, and pluripotent stem cells) [2]. The cardiac differentiation potential of adult, multipotent, stem cells found in fetal and adult tissues, however, is controversial [3, 4]. This has been attributed to the limited plasticity of adult stem cells, which precludes their differentiation into functional cardiomyocytes. The only adult stem cells that clearly have the potential to differentiate into beating cardiomyocytes are cardiac progenitor cells [5]. In addition, so far, only pluripotent stem cells have been shown *in vitro* to efficiently differentiate into spontaneously contracting cardiomyocyte-like cells [6–9]. 

Pluripotent stem cells have nearly unlimited self-renewal capability *in vitro* and have the ability to differentiate into all three germ layers and thus, in principle, can give rise to all cell types of the human body [10]. Since the first description of the isolation and characterization of human embryonic stem cells (hESCs) from donor blastocysts [11], there have been reports of differentiation of hESCs to functional cardiomyocytes by multiple differentiation methods. Recent breakthroughs in the field of induced pluripotent stem (iPS) cell technology have demonstrated that human iPS cells may provide an additional source for *in vitro* differentiated cardiomyocytes, sharing similarities with their hESC-derived counterparts [12–14]. Currently, cardiomyocytes can be differentiated from pluripotent stem cells by (1) spontaneous embryoid body (EB) differentiation in suspension, (2) coculture with mouse endoderm-like cells (END-2 cells), or (3) guiding the cardiac differentiation with defined growth factors either in suspension or in monolayer culture [15].

Owing to the cardiac phenotype and the functional properties of the pluripotent stem cell-derived cardiomyocytes, they can offer more physiologically and clinically relevant reproducible human cell models than presently available. *In vitro* differentiated cardiomyocytes may serve as models to study early events of human cardiogenesis and have the potential to be used in pharmaceutical drug discovery and safety toxicology. Animal models currently widely used in drug metabolism and toxicity studies are not fully reliable predictors of human responses because of basic physiological differences between species such as remarkably faster beating rate of the mouse may override the effects of arrhythmias which would be severe for human. It is anticipated that pluripotent stem cell-derived cardiomyocytes will be important *in vitro* tools for drug safety test which may drastically facilitate drug development and generation of safer drugs. In the field of cardiac regenerative repair, stem cell-derived cardiomyocytes would facilitate the discovery of small molecules promoting cardiomyocyte differentiation to be used for the activation of endogenous cardiac stem cells in clinical settings [15]. There is an urgent need to standardize and validate assays involving pluripotent stem cell-derived cardiomyocytes as well as to compare these cardiomyocytes with established *in vitro* and *in vivo* models in order to effectively determine the capabilities and limitations of the new models in making accurate predictions of the cardiac safety profile of new potential drugs during the development [15, 16]. The ability to reprogram adult cells to pluripotent stem cells and to genetically manipulate stem cells presents opportunities to develop models of human diseases [17, 18]. Until today, many severe diseases have been studied in animal models, particularly using transgenic mice. Although mouse models can provide valuable information, differences between human and mouse physiology limit the applicability of the results. iPS cells derived from patients suffering from various cardiac diseases or disorders, such as channelopathies or congenital heart disease [19, 20], are anticipated to become important tools for studying the mechanisms underlaying the disease pathogenesis and investigations of new treatment opportunities. However, it remains to be demonstrated that* in vitro* generated patient-specific iPS cell-derived cardiomyocytes actually recapitulate the appropriate disease phenotype observed in the adult heart.

Adult human cardiomyocytes are considered terminally differentiated cells. Although a small percentage of the cardiomyocytes may have proliferation capacity, it is not sufficient to replace injured or dead cardiomyocytes, for example, following myocardial injury. In recent years, the stem cell technology has raised hopes for new treatments for cardiac tissue damage with limited regenerative capacity. The possibility to apply pluripotent stem cell-derived cardiomyocytes to repair damaged myocardium has been demonstrated in preclinical studies to be a feasible approach although many hurdles remain to be solved before these developments can be translated to the clinic [21–28]. In brief, the obstacles to be solved include the generation of purified cell preparations that eliminate the risk of teratoma formation, defining the optimal timing for cell transplantation after myocardial infarction, the appropriate level of maturation of the cell preparation, dose and mechanism of delivery to facilitate successful engraftment, minimizing graft cell death following transplantation, and avoiding immune rejection of the graft [16]. Additionally, concerns remain that currently available pluripotent stem cell-derived cardiomyocyte preparations include myocytes with nodal, atrial, and ventricular type of action potential (AP) properties [8, 9]. This electrophysiological heterogeneity represents both an opportunity and a challenge to the application of stem cell-derived cardiomyocytes to cardiac repair. An enriched preparation of nodal cells would be of potential use in the formation of a biological pacemaker [29]. On the other hand, nodal cells would be needed to exclude from cardiomyocyte preparations for infarct repair, as their sustained pacemaking activity and unique neurohormonal responsivity could exacerbate the already elevated risk of arrhythmias [30]. 

Efficient methods to induce differentiation to cardiomyocytes that generate homogenous populations of cardiomyocytes of adequate quality, reproducibly, and in large quantities are a prerequisite for any of these applications. Future cell therapies will also require defined cardiomyocyte production protocols fulfilling the regulatory requirements. In this paper, we describe the developmental progression from a pluripotent stem cell state to cardiomyocyte and provide an overview of protocols for cardiac differentiation of pluripotent stem cells as well as enrichment strategies of cardiomyocytes so far available.

## 2. Lessons from Heart Development

**Figure 1:**
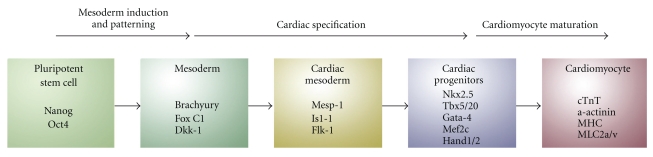
Diagram depicting sequential steps required for obtaining cardiomyocytes from pluripotent stem cells. Early mesoderm differentiates via cardiac mesoderm and committed cardiac progenitors further to functional beating cardiomyocytes. Typical markers for each step are indicated.

The earliest events of organogenesis during embryonic development are the formation of the heart. Although the knowledge regarding the molecular mechanisms that govern cardiogenesis in humans is still in its infancy, experimental animal models have been of great value for identifying various molecular events and a number of key regulators operating under the different stages of the early cell commitment process during cardiogenesis. Studies in mice and chick embryos have demonstrated that the heart tissue is composed of three major mesoderm-derived cell lineages: the cardiac myocyte, the vascular smooth muscle, and the endothelial cell lineages. Soon after gastrulation, a few days after fertilization, the three embryonic layers form, the endoderm, the ectoderm, and the mesoderm. The primitive streak is formed from primitive endoderm and is the origin of many tissues. Cardiac progenitors form in the posterior primitive streak [31]. Four main steps are required to generate cardiomyocytes from pluripotent stem cells: (1) formation of mesoderm, (2) the patterning of mesoderm toward anterior mesoderm or cardiogenic mesoderm, (3) formation of cardiac mesoderm, and (4) maturation of early cardiomyocytes. The induction of pluripotent stem cell differentiation to cardiac fate following these steps can be characterized by the expression of transcription factors, such as T/Brachyury for primitive streak mesoderm, Mesp-1 for cardiogenic mesoderm, and Nkx2.5, Tbx5/20, Gata-4, Mef2c, and Hand1/2 for cardiac mesoderm [32–36]. Mesp-1 is thought to induce an epithelial-mesenchyme transition in the epiblast and to bind directly to regulatory DNA sequences in the promoters of many members of the core cardiac regulatory network, including Nkx2.5 thereby promoting development of mesoderm precursors of the cardiovascular lineage as well as repressing the expression of key genes regulating other early mesoderm derivatives [37, 38]. Maturing cardiomyocytes can be identified by the expression of cardiac structural proteins such as *α*-actinin, *α*-myosin heavy chain (*α*-MHC), or the cardiac isoform of Troponin-T (cTnT). By initiating the complex myocardial cross-regulatory network, these factors are believed to be involved in morphogenic events leading to the formation of the heart.  Figure 1 illustrates various steps in the differentiation of pluripotent stem cells to cardiomyocytes and indicates the possible cell populations that may be available for isolation and expansion.

Cardiac development is a dynamic process that is tightly orchestrated by the sequential expression of multiple signal transduction proteins and transcription factors working in a combinatory manner. A number of signaling pathways and growth factors have been implicated in the development of specialized cardiac subtypes, and among the most studied are Wnts/Nodal, BMPs, and FGFs [39–42]. Since these factors work optimally during certain time window and, in some instances, antagonize cardiogenesis during other windows, the timing of the addition to guide the pluripotent stem cells in the desired differentiation direction must be carefully optimized. 

In addition, microRNAs (mir) have been recently identified as major contributors to the differentiation process [43, 44]. MicroRNAs are 22-23 nt long ssRNA involved in gene regulation and capable of inhibiting initiation of translation and inducing mRNA degradation. The heart expresses miR-1, miR-133, miR-206, and specifically miR-208 [45, 46]. Furthermore, miR-143 and -145 also appear important for cardiomyogenesis [47]. A recent study investigated expression of miR-1 and miR-133 in mESCs and hESCs and compared their potential to induce Nkx2.5 in embryoid bodies and their influence on mesodermal differentiation. miR-1 increased the proportion of beating aggregates and thus appears to greatly enhance cardiac phenotype. On the contrary, miR-133 promotes also early mesoderm differentiation but inhibits further cardiac commitment [48]. Interestingly, both of these miRs are coexpressed from the same cluster [49]. Identification of miRs regulating the cardiac differentiation will offer another route for guiding the differentiation of cardiomyocytes.

The first step (i.e., mesoderm induction) in cardiac differentiation from pluripotent stem cells has been well characterized. Numerous studies have demonstrated that Wnts, BMPs, and transforming growth factor (TGF) *β*-family member Nodal (or Activin A as a substitute of Nodal) efficiently induce mesoderm [50, 51]. Although some of the mechanisms that control for the next two steps in cardiac differentiation (i.e., patterning to cardiogenic mesoderm and the formation of cardiac mesoderm) have been characterized in experimental animal embryos, knowledge about how these mechanisms might be applied to pluripotent stem cell cardiogenesis is still lacking. Nodal and Wnt inhibition have been found to regulate the formation of cardiomyocytes in xenopus and chick embryos [52–54] and seem to be important for mouse ESC differentiation to cardiomyocytes [55, 56]. Dickkopf-1 (Dkk-1) is often employed as a Wnt antagonist at this stage of differentiation protocols [57]. Another important signaling pathway is mediated by the transmembrane receptor Notch, which has been shown to induce the expression of a combination of the growth factors Wnt5a, BMP6, and Sfrp1, which increase the amount of cardiac progenitors form an ESC-derived mesoderm subpopulation [58]. The last step in the differentiation stages to cardiomyocytes is the differentiation of committed cardiac progenitors to beating cardiomyocytes, a process that often occurs spontaneously *in vitro* and is poorly understood but might be controlled by factors such as Wnt11 [56]. Taken together, very little is known about the two crucial steps in cardiogenesis from pluripotent stem cells that is, promotion of mesendoderm to form committed cardiac mesoderm and factors to give rise to cardiomyocytes.

## 3. Cardiac Differentiation of Pluripotent Stem Cells

**Figure 2:**
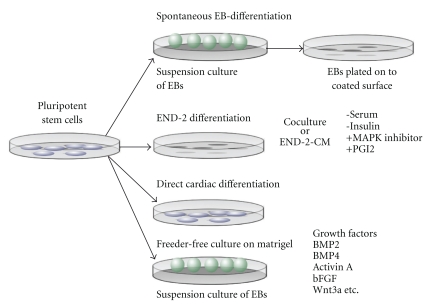
Schematic overview outlining differentiation approaches currently used for cardiomyocyte differentiation from pluripotent stem cells. *The embryoid body* approach has, thus far, been the most utilized way to obtain beating cardiomyocytes from pluripotent stem cells and the formation of three-dimensional cell aggregates initiates and facilitates the differentiation process. Generally, cells are transferred to suspension cultures, or in order to obtain more stable and reproducible cell aggregates, the embryoid bodies are formed using the hanging-drop or the forced aggregation method. *Using the END-2* approach, cardiomyocyte differentiation is triggered either by coculture of pluripotent stem cells, with END-2 cells or by embryoid body formation in suspension culture using END-2 conditioned medium. The depletion of serum and insulin has been shown to facilitate cardiogenesis in this approach, and it can be further enhanced by inhibiting p38 MAPK pathway by a specific inhibitor or by adding prostaglandin I_2_. *In the guided differentiation* approach, undifferentiated pluripotent stem cells are cultured under feeder cell-free conditions or in suspension culture after embryoid body formation. Cardiac differentiation is induced with various growth factors, such as BMP2, BMP4, Activin A, bFGF, and Wnt3a.

Table 1 and Figure 2 summarizes the differentiation approaches currently used for cardiomyocyte differentiation from pluripotent stem cells.

### 3.1. Embryoid Body Formation and Spontaneous Cardiomyocyte Differentiation

The initial observation that hESCs could mature into spontaneously contracting cardiomyocyte-like cells was reported ten years ago when hESCs were cultured in suspension and formed three-dimensional aggregates called embryoid bodies [6, 59]. Within the embryoid body, derivatives of the three germ layers (ectoderm, endoderm, and mesoderm) develop spontaneously. Within these mixed population of cells contracting areas with functional properties of cardiomyocytes can be detected. Cardiomyocytes are one of the first cell types induced from pluripotent stem cells in embryoid bodies, where cell to cell interactions stimulate the expression of markers for mesodermal and early cardiac cell lineages [60]. Typically, the embryoid bodies are plated on a matrix-coated tissue culture plate for further differentiation, and within a few days after plating, contracting outgrowths with cardiomyocyte characteristics can be observed [10]. As for human iPS cells, cardiomyocyte induction using embryoid body method was reported for the first time in 2009 [12]. The spontaneous differentiation of cardiomyocytes from pluripotent stem cells in embryoid bodies is considered to be rather inefficient usually under 10% and is highly cell line dependent [6]. 

Cardiomyocyte induction in the embryoid body-based differentiation system has also proven quite variable partly due to the heterogeneity among the aggregates that may, for example, differ in size and morphology. One strategy to address this issue was by adoption of the hanging drop method generally used with mESC [61]. However, this method has not been very successful with hESCs regardless of more consistency in the embryoid body formation. As an alternative, the forced-aggregation method was introduced, in which a defined number of dissociated hESCs were centrifuged into a round-bottomed ultra-low attachment 96-well plate which allowed for better control of the embryoid body size [62]. Although this method was quite reproducible, as well as considered fairly practical and efficient, the forced-aggregation method was not originally developed for cardiomyocyte differentiation [62]. 

At that time when embryoid body cardiac differentiation method was introduced, limited information regarding the mechanisms underlying cardiogenesis in human pluripotent stem cells was available. Later, it has become evident that the differentiation process can be manipulated by the addition of growth factors, morphogenes, or by transgenic modifications to direct the pluripotent stem cells toward the cardiac cell fate. Spontaneous differentiation of hESCs, cultured as aggregates or embryoid bodies, has been shown to be enhanced by demethylating agent 5-aza-deoxycytidine [61]. By using the embryoid body cardiac differentiation method with 5-aza-deoxycutidine, between 8% and 70% of the embryoid bodies showed beating areas, and 2% to 70% of the beating areas consisted of cardiomyocytes [61]. Low oxygen tension has been shown to increase the number of cardiomyocytes from hESCs in bioreactors. Cardiac differentiation at 4% oxygen increases the total cell number by 30%–47% as well as some cardiac markers when compared with 20% oxygen [63]. Electrical stimulation with EB differentiation method has also been shown to enhance cardiac differentiation of hESCs through mechanisms associated with the intracellular generation of ROS [64]. Although many other approaches have been used to generate cardiomyocytes from pluripotent stem cells, the embryoid body formation in suspension cultures remains widely applied method to induce cardiomyocyte differentiation largely due to its simple and inexpensive nature.

### 3.2. Coculture of Pluripotent Stem Cells with Cardioinductive Cell Types

Another approach for cardiac differentiation was inspired by developmental studies indicating the critical role of anterior endoderm in the cardiac induction of adjacent mesodermal structures [65–67] and has been widely applied to induce cardiomyocyte differentiation *in vitro*. The method is based on coculture of pluripotent stem cells with a visceral endoderm-like cell line (END-2), derived from mouse P19 embryonal carcinoma (EC) cells, which results in the formation of beating clusters of cells that also display characteristics of cardiomyocytes [9, 68]. The cardiac differentiation efficiency of the method can be enhanced in the absence of serum and with ascorbic acid. Ascorbic acid has been shown to upregulate late-stage markers of cardiogenesis [69]. Others as well as our group has found that this differentiation method works comparably well with iPS cell lines [70]. An END-2 conditioned media (END-2-CM) system has also been demonstrated to induce robust differentiation of cardiomyocytes in hESC-derived embryoid bodies [71–73]. Cardiomyocyte differentiation from pluripotent stem cells occurs within 12 days of co-culture with END-2 cells. Based on cardiomyocyte phenotype and electrophysiology, the majority of pluripotent stem cell-derived cardiomyocytes resemble human fetal ventricular cardiomyocytes [9]. However, the cardiac differentiation efficiency from standard END-2 co-culture experiments is usually fairly low. 

Based on the knowledge from developmental biology, the cardioinductive signals are thought to originate from direct cell to cell contact and/or secreted factors from the END-2 cells which stimulate the presence of embryonic endoderm [71]. Although the exact mechanism of END-2 cardiac induction is still unclear, the transcriptome and secretome of END-2 cells have been described [74, 75]. Cardiac induction by END-2-CM can be at least partly mimicked by insulin depletion [72], inhibition of p38 MAPK [66], and addition of prostaglandin E [73]. END-2 cells have been shown to clear insulin from the medium and to secrete more PGI_2_ than any other type of mouse cells which lack the cardiac inductive effect [72, 73]. Interestingly high concentrations of insulin appear to favor differentiation to neuroectoderm, and blocking p38 MAPK further enhances cardiac differentiation [71]. p38 MAPK signaling has been found to be highly active for the neuroectoderm formation and to be inhibitor of cardiomyogenesis, and thus blocking of this pathway may favor meso/endoderm differentiation [71]. These modifications to the END-2 co-culture protocol have been reported to result in preparations of 20%–25% cardiomyocytes. Identifying further cardiomyocyte inducing factors from the END-2 cells may provide opportunities to develop defined and more efficient process of differentiation for the induction of stem cells to cardiomyocytes and cardiac progenitors. Recently, a method for inducing maturation by replating the cardiomyocytes initially differentiated in co-culture with END-2 cells on to fresh END-2 cells followed by a short three-dimensional culturing step was described [76]. The method enables cardiomyocyte maintenance for up to 1 year with increasing maturation in terms of their electrophysiological properties as well as increase in the number of pacemaker cells. 

### 3.3. Guided Cardiomyocyte Differentiation with Specific Factors

The signaling pathways regulating the cardiogenesis can be recapitulated in cell culture by the addition of specific growth factors such as FGFs, BMPs, and Wnts. Several studies have shown that combinations of BMP4, Wnt3a and Activin A induce gastrulation-like events and meso-/endoderm development in pluripotent stem cells [77–79]. At least a part of the cardioinductive activity of anterior endoderm is mediated by growth factors belonging to the TGF*β*-superfamily [80, 81], and a guided cardiac differentiation involving two TGF*β*-family members, Activin A and BMP4, has been described [27]. In this approach, based on a high-density monolayer, pluripotent stem cells are cultured in a feeder cell-free system and cardiomyocyte differentiation is induced by a defined serum-free medium, supplemented sequentially with BMP4 and Activin A. The growth factors are then removed, and the cells are maintained in serum-free medium for an additional 2-3 weeks in the absence of exogenous growth factors. Spontaneously contracting areas are generally observed approximately 10 days after induction with Activin A, and enzymatically dissociated preparations at three weeks after induction typically consist of >30% cardiomyocytes [27]. Both hESCs and iPS cells have been differentiated to cardiomyocytes by using this approach [17, 27]. 

Another protocol also involves TGF*β*-family molecules but also exploits the important roles of canonical Wnt signaling in cardiogenesis. Canonical Wnt signaling exerts stage-dependent effects on cardiac differentiation: it is required for mesoderm induction but must be inhibited later for the induction of precardiac mesoderm [55, 56]. Based on this information, a guided embryoid body differentiation protocol was developed involving induction of a primitive streak-like population in addition to formation of cardiac mesoderm with Activin A, BMP4, bFGF, and VEGF followed by cardiac specification with the Wnt inhibitor, Dkk-1 [57]. This protocol has been described to produce populations consisting of ~40%–50% cardiomyocytes. The protocol was further enhanced by sorting the differentiating cultures for an early cardiovascular progenitor based on expression of the Flk-1 (also known as KDR). The early differentiating embryoid bodies include two KDR^+^ populations: an early hemangioblast population (i.e., hematopoietic and endothelial progenitors) and another multipotent cardiovascular progenitor population that can be distinguished at 5-6 days after induction based on their differential expression of KDR and the stem cell marker c-kit [82]. In particular, if KDR^high^/c-kit^+^ cardiovascular progenitors are selected by fluorescence-activated cell sorting (FACS) at this timepoint and then replated in monolayer cultures, they subsequently differentiate into highly enriched preparation of cardiomyocytes, endothelial cells, and smooth muscle cells. Mesendoderm formation has also been induced by Wnt3A, an activator of the canonical Wnt/*β* catenin signaling pathway in cardiac differentiation protocol [60]. In this protocol, hESCs were transiently treated with Wnt3A or BMP4, in the initial steps of embryoid body formation, and then subsequently decreased the amounts of serum and insulin in the culture medium [60].

Another recent report described the cardiac differentiation of pluripotent stem cells with BMP2, acting in a combinatorial manner with Wnt3, whose expression was triggered by the morphogen, to drive pluripotent stem cells toward an early mesodermal and cardiogenic fate *in vitro*. BMP2-induced Oct-4^+^ and SSEA-1^+^ cells give rise to endo/mesendodermal cells secreting cardiogenic factors, further directing the cell fate toward a cardiac phenotype when plated on MEFs releasing bFGF [5]. The addition of PDGF or VEGF to BMP2-induced SSEA-1^+^ cells cultured on MEFs further directs the fate of the cells toward a smooth muscle and endothelial phenotype.

Chemical biology offers alternative means for discovering novel cellular signaling molecules mediating pluripotent stem cell cardiogenesis. As a consequence, high-throughput molecular screening technology has been exploited to search for compounds with the potential to induce cardiomyogenesis *in vitro*. To date, a few studies have published results from such screening approaches, describing the identification of novel small molecules that appear to stimulate the generation of cardiomyocytes from pluripotent stem cells, including cardiogenols, ascorbic acid, isoxazolyl-serines, sulfonyl hydrazones, and DMSO [69]. All of these molecules were identified based on their ability to upregulate late-stage markers of cardiogenesis. While some clearly have many effects on cells such as DMSO and ascorbic acid, others are probably more selective. Whether these factors have direct role in cardiogenesis or if they stimulate certain other cell populations which in turn activate cardiac development remains to be determined.

### 3.4. Cardiac Progenitor Cells

A growing evidence suggest that all three major cardiac cell lineages may arise from a common multipotent cardiovascular progenitor cell population originating in primitive streak and displaying specific expression of markers such as Flk-1, c-kit, and Isl-1 [5, 57, 82–84]. These cardiovascular progenitor cells have recently also been identified in early pluripotent stem cell derivatives [5, 57]. Due to their proliferative capacity in culture, these cell populations would be ideal for upscaling *in vitro*. Furthermore, these early cardiac restricted precursor cells would allow for the establishment of progenitor cell based *in vitro* models for uncovering the early events of cardiogenesis. Strategies that might improve cardiomyocyte yields through stimulation of proliferation of committed progenitors might also be valuable. Information in regard to the signals that stimulate the replication of committed progenitors is still lacking; however, activation of canonical Wnt signaling has been demonstrated to expand the pool of Nkx2.5^+^, and Isl1^+^ early cardiac progenitors [39, 54, 60, 85], and the activation of the Notch pathway in immature cardiomyocytes has been shown to prolong their period of replicative competence [86, 87]. Other suggested mediators that might hold committed progenitors in a proliferative state are the GSK-3 inhibitor BIO [88], p38 MAPK inhibition [89], and the PI3K/Akt pathway [90].

## 4. Enrichment Strategies of Stem Cell-Derived Cardiomyocytes

Despite the tremendous progress in the development of current cardiac differentiation protocols, none of the currently available protocols results in homogenous populations of cardiomyocytes. One of the challenges over the last years has been to develop robust isolation techniques that allow scalable purification of cardiomyocytes and specific cardiac subtypes. The most straightforward approach is mechanical isolation based on manual dissection of the spontaneously contracting cardiac cells. The microdissected cells from even relatively low-purity cell population can include up to 70% cardiomyocytes [6, 9, 24]. Another fairly exploited approach presenting significant improvements to the enrichment process is Percoll density gradient centrifugation, which takes advantage of the unique buoyant properties of cardiomyocytes. Percoll centrifugation can result in a three- to sevenfold enrichment of cardiomyocytes and has been applied for enzymatically dispersed cells from embryoid bodies and for guided differentiation protocols [7, 22, 27, 77]. However, these enrichment protocols have disadvantages in regard to insufficient purity, fairly labor intensive procedure, and lack of scalability. 

A recent study utilized an endogenously expressed surface marker, ALCAM, to isolate cardiomyocytes from a mixed population of differentiated cells [78]. This approach, however, has proven rather challenging for purifying cardiomyocytes from pluripotent stem cells due to the limitation of available cardiomyocyte-specific cell-surface markers. Another recent study demonstrated that a fluorescent dye labeling mitochondria could be used to selectively mark human pluripotent stem cell-derived cardiomyocytes and, subsequently, utilized in the enrichment of cardiomyocytes (>99% purity) by fluorescence-activated cell sorting [91]. Other recent reports have demonstrated the guided differentiation and subsequent isolation of an early population of cardiovascular progenitors, expressing Oct4, SSEA-1, and Mesp1. The isolation of the progenitors was based on cell sorting using an anti-SSEA-1 antibody allowing separation of cells expressing mRNAs and proteins encoding mesodermal and cardiac markers [5, 92]. 

To date, the highest levels of cardiac purity have been obtained using genetic selection techniques. In this strategy, undifferentiated pluripotent stem cells are genetically modified to carry either a reporter gene, usually a green fluorescence protein (EGFP) or mammalian selection gene (e.g., antibiotic resistance) under the transcriptional control of a cardiac-specific promoter. The transgenic cells are then induced to differentiate and selected based on activation of the cardiac-specific promoter. While the major disadvantage of this approach is that it entails the usual risks of genetic modification (e.g., insertional oncogenesis), it is capable of a significant degree of cardiac enrichment [79, 93]. Genetic selection based on activation of either the human *α*-MHC [94, 95] or MLC2v [96, 97] promoters has been shown to generate populations of >90% human pluripotent stem cell-derived cardiomyocytes. To sum up, most of the recently published cardiomyocyte enrichment studies demonstrate the advantages of using the transgenic strategy based on cardiac-specific drug resistance selection, either alone or in a combination with the reporter gene approach [73, 98, 99]. The influence of other cell types on cardiac differentiation is one aspect still to be further studied to determine at which state purification would be optimal [100]. While generally considered useful for* in vitro* applications, improvements and/or alternative strategies need to be developed to overcome the additional hurdles before introducing genetically modified cells into clinical cell therapy. 

## 5. Characteristics of Pluripotent Stem Cell-Derived Cardiomyocytes

During recent years, a number of published studies have described the basic characteristics of pluripotent stem cell-derived cardiomyocytes. In these reports, cell analysis has been based on the expression of specific molecular markers for cardiomyocytes, structural architechture, and functionality. Although substantial heterogeneity has been reported, in general, 30%–60% of the cells in isolated beating areas display markers and other features of cardiomyocytes [6, 101–103]. Pluripotent stem cell-derived cardiomyocytes usually have less defined rod shape compared to their mature adult counterparts. In addition, pluripotent stem cell-derived cardiomyocytes display multinucleation at a very limited frequency (<1%) [101] compared to adult human cardiomyocytes (20%). At the ultrastructure level pluripotent stem cell-derived cardiomyocytes show clearly idenfiable sarcomeres with A, I, and Z bands and intercalated discs with gap juctions and desmosomes, and these cells share similarities with adult cardiomyocytes although the myofibrillar and sarcomeric organization indicate an immature phenotype in the stem cell-derived population [6, 61, 101, 102]. In the vicinity of the sarcomeres, mitochonrdia are also present.

On a molecular level, several markers expressed by cardiomyocytes are also expressed by pluripotent stem cell-derived cardiomyocytes, including transcription factors, structural proteins, hormones, ion-channels, and tight junction proteins [6–9, 61, 67, 103]. Expression of early cardiac-specific transcription factors involved in cardiogenesis such as GATA-4, Nkx2.5, Isl-1, Tbx-5, Tbx-20, and Mef2c is generally observed also in the pluripotent stem cell-derived cardiomyocytes [6, 7, 61, 67, 103]. In addition to structural proteins including sarcomeric proteins *α*-actinin, cardiac troponins T, and I, sarcomere myosin heavy chain (MHC), atrial- and ventricular myosin light chains (MLC-2A and MLC-2V), desmin, and tropomyosin, gap junction proteins are also expressed [7–9]. The presence of other cardiac and muscle-specific proteins including atrial natriuretic peptide (ANP), creatine kinase-MB, and myoglobin has also been described in several reports [7, 104]. Troponin complex is located on the thin filament of striated muscles and regulates muscle contraction in response to alterations in intracellular calcium ion concentrations.

More important, however, are the functional characteristics of the cells, and different pharmacological and electrophysiological approaches have been used to examine these properties. As in mature cardiomyocytes, the trigger for contraction in pluripotent stem cell-derived cardiomyocytes is a rise in intracellular calcium [9, 105]. However, the regulation of intracellular calcium handling has been shown to differ between pluripotent stem cell-derived cardiomyocytes and mature adult cardiomyocytes, most likely due to the apparently immature sarcoplasmic reticulum which has been suggested to be caused by lack of expression of phospholamban and calsequestrin, which are two of the main intracellular calcium handling proteins [105–107]. One major advantage of cardiomyocytes derived from pluripotent stem cells is that they can be maintained in culture for extended time periods even months without losing their spontaneous contractile capacity. Although pluripotent stem cell-derived cardiomyocytes show unambiguous cardiac-type action potentials (AP), these cells exhibit comparatively immature AP parameters such as automaticity, a slower AP upstroke, and a relatively depolarized maximum diastolic potential [8, 108]. Several studies have demonstrated that pluripotent stem cell-derived cardiomyocytes exhibit spontaneous contractile activity that could be modulated by drugs such as isoproterenol and carbachol, and thus these cells respond to alpha/beta-adrenergic- and muscarinic stimuli further indicating that the cells express specific surface membrane receptors coupled to a signaling pathway that activate ion channels, membrane transporters, and myofilament proteins [6, 7, 103]. In contrast to a typical mature cardiomyocyte characteristic increase in contraction amplitude in response to increased stimulation in the (i.e., the force-frequency relation) in the adult myocardium, pluripotent stem cell-derived cardiomyocytes have been reported to respond in the opposite manner and display negative force-frequency relations [104]. 

In general, cardiomyocyte induction from pluripotent stem cells results in mixtures of ventricular-like, atrial-like, and pacemaker-like cells defined by intracellular electrophysiological measurements of action potentials (APs). As shown in voltage-clamp studies, pluripotent stem cell-derived cardiomyocytes exhibit expected ionic currents, such as fast sodium current, L-type calcium current, pacemaker currents, as well as transient outward and inward rectifier potassium currents [9, 108] Interestingly, different differentiation protocols seem to affect the ratios of these cardiac cell types. While most differentiation protocols based on embryoid bodies/cell aggregates result in more or less equal numbers of ventricular- and atrial-like cells, cardiac induction by END-2 co-culture generally results in cell population where the majority of the cardiomyocytes display ventricular-like phenotype based on morphological and electrophysiological parameters [9].

In summary, based on the gene expression profile and the structural-, electrophysiological-, and pharmacological properties of the pluripotent stem cell-derived cardiomyocytes they possess immature phenotype and appear to have an immature sarcoplasmic reticulum function. The cardiac phenotype of iPS cell-derived cardiomyocytes seems to be comparable to that of hESC-derived cardiomyocytes [17]. Pluripotent stem cell-derived cardiomyocytes have been shown to mature over time in culture but without carrying through to full maturity. Thus, additional research is needed to find novel strategies to mature pluripotent stem cell-derived cardiomyocytes *in vitro*. Nevertheless, even if some functional properties of pluripotent stem cell-derived cardiomyocytes apparently differ from the mature cells present in adult myocardium the *in vitro* generated cardiomyocytes have a basal functionality and can still provide a useful model for molecular cardiology.

## 6. Conclusion and Future Perspectives

Cardiac differentiation is a dynamic process consisting of complex signaling network, and although various signaling pathways and growth factors have been implicated in the development of specialized cardiac subtypes, only limited information about the mechanisms underlaying cardiogenesis in human pluripotent stem cells is currently available. Thus, the differentiation of pluripotent stem cells toward cardiomyocytes is still poorly defined compared with the other cell fates. A number of differentiation protocols have been described to generate cardiomyocytes from pluripotent stem cells. Collectively, various cardiac differentiation studies demonstrate how the exposure of various growth factors to pluripotent stem cells, at an accurate timing and dose, is essential for directing the differentiation process from early mesendoderm via mesoderm towards a more specific cardiac fate. Despite the recent advances in the cardiac differentiation protocols, in most settings the cardiomyocyte differentiation is uncontrolled and inefficient remaining less than 10% [69]. Most of the iPS cell lines generated to date are subject to viral integration that may have an impact on the cardiogenesis of these cells. However, human iPS cells appear to have a cardiac potential highly comparable to hESCs, and iPS cells can be differentiated into cardiomyocytes using similar differentiation protocols [17]. Based on the variations of the protocols used and the efficiency at which pluripotent stem cells differentiate to cardiomyocytes, it appears that pluripotent stem cell lines including both hESC and iPS cell lines behave quite differently, implicating that the specific pluripotent stem cell line utilized might affect the final result. Thus, each pluripotent cell line may require a specific differentiation protocol for efficient induction of cardiogenesis further complicating the overall assignment. Evidently, a more in-depth understanding of these mechanisms will improve the yields of pluripotent stem cell-derived cardiomyocytes for large-scale and clinical application.

The stem cell-derived cardiomyocytes described to date, generally resemble fetal cardiomyocytes exhibiting immature functional and structural characteristics compared to adult cardiomyocytes and mature only slowly in cell culture. Furthermore, the differentiated cells are mixed populations of noncardiac cells and cardiomyocytes with several subtypes (e.g., ventricular-, atrial-, and nodal type) and maturation stages. Currently, we are also lacking efficient ways to guide the cardiac subtype differentiation as well as to isolate cardiomyocytes and subtypes of cardiomyocytes from the heterogeneous pool of differentiating cells. Thus, efficient enrichment strategies for cardiomyocytes as well as different cardiac sub-types are urgently needed. A recent study provided a strategy for the enrichment of cardiomyocytes and in particular, the generation of a specific subtype of cardiomyocytes, nodal-like cells, without genetic modification. They utilized the activator of Ca^2+^-activated potassium channels of small and intermediate conductance (SKCas) on embryonic stem cells leading to induction of cardiac mesoderm and cardiomyocyte specification resulting in a strong enrichment of pacemaker-like cells [109]. 

Most of the current cardiac differentiation methods produce beating aggregates, but for many purposes, monolayer cardiac differentiation would be optimal. However, it has been shown that cell-to-cell interactions in the embryoid body and aggregate structures stimulate the expression of markers for mesendoderm and early cardiac cell lineages, which argues in favor of the three-dimensional cell aggregates for optimal pluripotent stem cell differentiation to cardiac myocytes [60]. Nevertheless, the current differentiation protocols produce cardiomyocytes that possess many promising capabilities and have wide-spread utility for basic research as well as pharmaceutical industry. In future, stem cell-derived cardiomyocytes are anticipated to have an enormous impact on the treatment of heart disease. The ability to induce iPS cells has raised the possibility to reprogram somatic cells to an alternative differentiated fate without first becoming a stem cell. Recently, it was shown that a combination of three transcription factors (Gata4, Mef2c, and Tbx5) reprogrammed cardiac or dermal fibroblasts directly into spontaneously contracting cardiomyocytes, expressing cardiac-specific markers [110]. These induced cardiomyocytes offer another important option to research, drug discovery and cell therapy towards cardiovascular regeneration.

## Figures and Tables

**Table 1:** Summary of prevalent cardiac differentiation methods.

Method description	Differentiation efficiency %	Stem cell type	Reference
Spontaneous embryoid body method	<10%	hESC hiPSC	Kehat et al. [6] Zhang et al. [12]
END-2 method Insulin depletion, PGI_2_, p38 MAPK inhibition	20%–25%	hESC hiPSC	Passier et al. [68], Graichen et al. [71] Freund et al. [70]
Guided differentiation method Activin A, BMP4	>30%	hESC hiPSC	Laflamme et al. [27] Takahashi et al. [17]
Guided differentiation method Activin A, BMP4, bFGF, VEGF, Dkk-1	40%–50%	hESC	Yang et al. [57]
